# How do we manage the gastrectomy for gastric cancer after coronary artery bypass grafting using the right gastroepiploic artery? Report of two cases and a review of the literature

**DOI:** 10.1186/1477-7819-5-54

**Published:** 2007-05-17

**Authors:** Yukiko Konishi, Koichi Suzuki, Hidetoshi Wada, Hiroshi Watanabe, Hiroyuki Ogura, Yuno Sugamori, Abul Hasan Muhammad Bashar, Katsushi Yamashita, Toshihiko Kobayashi, Teruhisa Kazui

**Affiliations:** 1 First Department of Surgery, Hamamatsu University School of Medicine 1-20-1, Handayama, Hamamatsu, 431-3192, Japan

## Abstract

**Background:**

Recently, the right gastroepiploic artery (RGEA) has been used in coronary artery bypass grafting (CABG) as an alternative arterial graft. Unfortunately, an increased incidence of gastric cancers has been reported after CABG using the RGEA. Handling of the RGEA during gastrectomy in these patients may cause lethal complications, which sometimes reduces the feasibility of curative dissection of lymph nodes at the base of the graft.

**Case presentations:**

We describe two cases of gastric cancer undergoing gastrectomy after CABG with the use of RGEA. To avoid the potentially fatal coronary event during gastrectomy, safe handling of the conduit including preparations for injuries and prevention of vessel spasm was performed in both cases, accompanied by an adequate monitoring of the systemic circulation. Intraoperative frozen section examination showed no lymph node metastasis around the graft in any of the cases; therefore, complete lymph node dissection at the base of the graft was not undertaken. No complications occurred during the operation. In addition to these two cases, twenty-four cases reported in the literatures were reviewed (a total of 26 cases). Ten early and 16 advanced gastric cancers were included. Among the 16 advanced gastric cancer cases, an alternative graft was employed in 8 due to the resection of an original graft to complete lymph node dissection. Mere handling of a graft often caused lethal complications suggesting that the operation should be completed by isolation of the graft. A pedicled graft harvesting via the ante-gastric route was popular. However, a skeletonized harvesting with resection of the pyloric branches of the RGEA would be better because this would interrupt the original lymph flow, which could eliminate the need for lymph node dissection and graft isolation. Among the 10 cases having early gastric cancers, 6 were found within 1.5 years after CABG. Early detection in these 6 cases was possible due to the use of gastric fiberscopic examination before and after CABG, which gave them opportunities to receive a less extensive operation such as endoscopic mucosal resection.

**Conclusion:**

Adequate intraoperative care as well as an optimal lymph node dissection considering the graft harvesting method at the first CABG leads to successful gastrectomy after CABG using the RGEA graft. Therefore, this operation should be carried out with careful management by both gastrointestinal and cardiovascular surgeons.

## Background

Recently, the right gastroepiploic artery (RGEA) has been used in coronary artery bypass grafting (CABG) as an alternative arterial graft [1,2], However, an increased incidence of gastric cancers has been reported after CABG using the RGEA. According to the report of Japanese association for coronary artery surgery , CABG was carried out in more than 0.1 million patients over a period of 7 years that ended in 2004, and the RGEA has been used in more than half of these patients. Spasm and injuries induced by handling of the graft during gastrectomy would cause critical coronary failure [3-8]. Appropriate strategy is, therefore, required to avoid risk while retaining the curative potential of the operation. We present two cases of gastric cancers who underwent gastrectomy after CABG using the RGEA with a review of 24 similar cases reported in the literature.

## Case presentation

### Case 1

**Figure 1:**
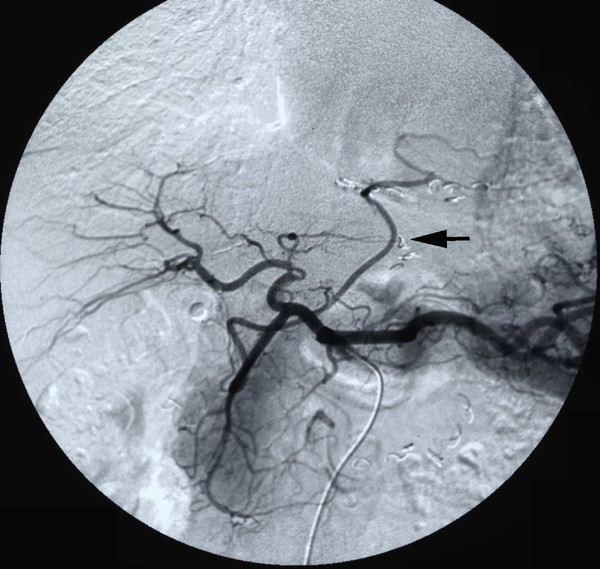
Preoperative angiography via celiac artery showed that the RGEA graft remained well patent.

The patient was a 76-year-old man. In November 1999, he underwent three- vessel CABG. A postero-lateral branch of the coronary artery was grafted by a pedicled RGEA. In July 2004, he presented to a private hospital because of tarry stool 5 years after CABG. By gastric fiberscopic examination, he was diagnosed to have an early gastric cancer (IIc) located in the lower anterior area of the stomach and was transferred to our hospital for gastrectomy in October. Preoperative angiography showed that the RGEA graft remained well patent (Figure 1).

**Figure 2:**
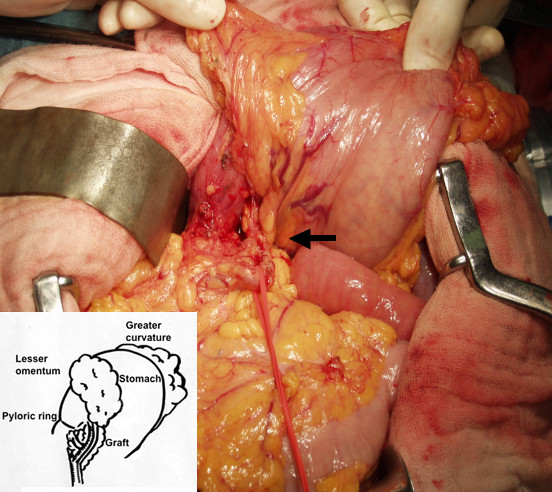
Intraoperative view of the RGEA. It was easily recognized on the left lobe of the liver accompanied by a vein and soft tissues although it was adherent to the posterior aspect of the lesser curvature of the stomach. An insert showed a scheme of the intraoperative view.

After an arterial catheter was inserted in the right femoral artery for an artificial circulation in preparation for a possible graft injury, an epigastric median incision was made. The RGEA was easily recognized on the left lobe of the liver accompanied by a vein and covered with soft tissues (Figure 2). Papaverine hydrochloride was sprinkled around the RGEA to prevent vessel spasm. Its adhesion to the posterior aspect of the lesser curvature of the stomach was dissected. We dissected around the No.6 lymph node station as far as we could do safely and easily. Intraoperative frozen section examination showed no lymph node metastasis around the graft; therefore, complete dissection of lymph nodes at the base of the graft was not undertaken. A distal gastrectomy with lymph node dissection was performed. A *Roux-en-Y *procedure was used for reconstruction. The pathological diagnosis was poorly differentiated adenocarcinoma (por1>tub2), (18 × 11 mm), sm2, n0 and clinical stage was determined as Stage Ia according to the General Rules for the Gastric Cancer Study [9]. No complications occurred during the operation. The patient is currently alive without any signs of recurrence 18 months after the operation.

### Patient 2

**Figure 3:**
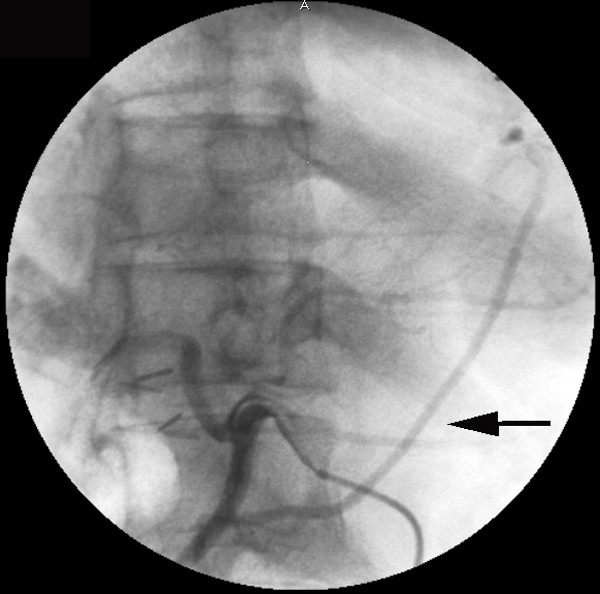
Preoperative angiography via celiac artery showed that the RGEA graft remained well patent.

The patient was a 64-year-old man. In November 2005, he underwent three-vessel CABG. The posterior descending branch of the coronary artery was grafted by a skeletonized RGEA. In February 2006, he presented to a private hospital because of epigastric pain. By gastric fiberscopic examination, he was diagnosed to have an early gastric cancer (IIa + IIb) located at the angle of the stomach and was transferred to our hospital for gastrectomy in April. Preoperative angiography confirmed the patency of the RGEA graft (Figure 3).

**Figure 4:**
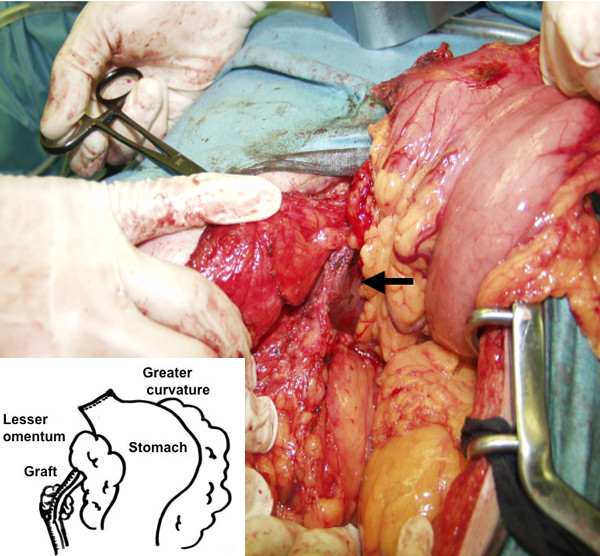
Intraoperative view of the RGEA. It was difficult to be found because it was covered with the surrounding tissues involving the wall of the stomach and the liver. Eventually, it was recognized by its pulsation on the duodenum. An insert showed a scheme of the intraoperative view.

After an arterial catheter was inserted in the right femoral artery for an artificial circulation to be used in case of a graft injury, an epigastric median incision starting below the umbilicus was made with care because the RGEA took an anterior route. The RGEA was covered with the surrounding tissues involving the wall of the stomach and the liver, making it difficult to be found (Figure 4). Papaverine hydrochloride was sprinkled around the RGEA to prevent vessel spasm. Eventually, it was recognized by its pulsation on the duodenum. Dissection around No.6 lymph node was carried out to the extent that was deemed safe. Since intraoperative frozen section examination revealed no lymph node metastasis around the graft, complete dissection of lymph nodes at the base of the graft was not undertaken. A distal gastrectomy with lymph node dissection was performed. A *Roux-en-Y *procedure was used for reconstruction. The pathological diagnosis was signet ring cell carcinoma (23 × 20 mm), m, n0 and clinical stage was determined as Stage Ia according to the General Rules for the Gastric Cancer Study [9]. There was no intraoperative complication. The patient is currently alive without any signs of recurrence 4 months after the operation.

### Features of the 26 cases (Table 1)

**Table 1:** Cases were ordered by the intervals after CABG

**Herv**	**Route**	**Terms**	**Pat**	**Op**	**Re-co**	**RGEA**	**Adh**	**Patho**	**pN**	**pT**	**Stage**	**No.6**	**Surv**	**Time**	**Ref**
ske	ante	3	well	gast	R-Y	pres	+	sig	n0	m	Ia	-	A	36	Case2
ped	ante	5	well	p res	-	pres	?	por1	n0	sm	Ia	ND	A	3	(4)
ped?	?	6	?	t gast	R-Y	pres	?	por2	n2	se	IV	?	A	12	(21)
ped	ante	10	well	gast	R-Y	pres	+	por	n1	sm2	Ib	+	?	?	(22)
ped	ante	10	?	gast	B-II	res	+	por	n1	se	IIIa	+	?	?	(16)
ske	ante	11	well	gast	B-I	res	?	por	n0	m	Ia	-	?	?	(17)
ske	ante	14	well	gast	R-Y	res	+	?	?	?	IV	ND	?	?	(10)
?	ante	18	well	gast	B-II	pres	++	tub1	n0	m	Ia	ND	A	10	(7)
ped	ante	18	well	gast	B-II	pres	+	pap	n0	sm2	Ia	-	A	10	(15)
ped	ante	20	well	gast	B-II	pres	?	?	n2	s1	IIIa	ND	D	8	(5)
ped	ante	21	?	c res	int	pres	-	tub2	n0	ss	Ib	-	?	?	(22)
ped	ante	21	well	gast	B-I	res	?	por	n2	ss	IIIb	+	?	?	(17)
?	retro	22	well	t gast	R-Y	pres	+	mod	n0	mp	Ib	-	A	2	(23)
ped	ante	22	?	gast	B-I	pres	+	tub2	n0	m	Ia	-	A	48	(24)
ped	ante	24	?	gast	B-II	res	?	por	n1	se	IIIa	+	?	?	(16)
ped	ante	26	stenosis	bypass	-	?	?	?	n3	s2	IV	ND	A	4	(4)
ped	ante	29	well	gast	B-I	pres	+	?	n0	m	Ia	ND	A	?	(18)
ped	?	32	stenosisgast		B-I	res	-	por	n1	ss	II	+	?	?	(19)
ped	ante	32	well	gast	?	res	?	?	n2	s2	III	?	D	16	(5)
?	?	36	obs	t gast	R-Y	res	?	?	?	?	?	?	?	?	(25)
ped	?	37	well	gast	B-II	pres	+	por	n0	ss	II	-	A	36	(26)
ped	ante	39	well	gast	B-II	pres	?	tub2	n0	mp	Ib	-	A	10	(4)
ped	ante	45	?	t gast	R-Y	pres	+	por	n1	ss	IV	?	?	?	(16)
ped	ante	63	well	gast	B-I	res	?	por	n2	se	IIIb	+	?	?	(17)
ped	retro	65	well	gast	R-Y	pres	+	por	n0	sm2	Ia	-	A	3	Case1
ped	ante	91	well	gast	R-Y	res	?	tub2	n0	m	Ia	-	A	17	(3)

Herv: harvesting method, ped: pedicled, ske: skeletonized, retro: retrogastric, ante: antegastric, Pat: patency, obs: obstruction, Op: operation, gast: partial (distal) gastrectomy, t gast: total gastrectomy, p res: partial resection, c res: partial resection of cardia, Re-co: re-construction, B-I: Billoth-I, B-II: Billoth-II, R-Y: Roux-en-Y, -: direct anastomosis, res: resection, pres: preserved, Adh: adhesion, +: positive, -: negative, Patho: pathology [9], pN: pathological lymph node metastasis [9], pT: pathological tumor invasion [9], No.6:Infrapyloric lymph node [9], +: positive, -: negative, ND: not dissected, Surv: survival, A: alive, D: dead, Time: survival time, Ref: references, ?: unknown

Twenty-one male and 5 female patients were included. Their ages ranged from 55 to 76 years (mean age, 66.4). The pedicled graft method was employed in 20 cases while the skeletonized graft method was used in only 3 cases. Antegastric route was popular and was used in 20 cases while the retrogastric route was used in only two cases. Intervals from CABG ranged from 3 months to 91 months (average, 27.7 months). In most cases, the graft was patent except the two cases of stenosis and one obstruction. The operative procedures and the reconstruction methods used were as follows: operation; partial gastrectomy in 19 cases, total gastrectomy in 5 cases, one partial resection and one bypass, reconstruction; Billroth-I in 7 cases, Billroth-II in 7 cases, Roux-en-Y in 9 cases and no reconstruction in two cases. Resection of the RGEA was performed for complete dissection of No. 6 lymph node in 10 cases while the RGEA was preserved in the remaining 16 cases. Adhesion was observed in 6 cases while was severe in two and mild in the remaining 4 cases. Six cases had metastasis to No. 6 lymph node. In two cases, both gastrectomy and re-grafting using the saphenous vein to the right coronary artery were performed simultaneously. In two cases, gastrectomy was carried out after re-grafting to the right coronary artery using a right internal thoracic artery and the right radial artery, respectively. The RGEA was replaced by the slpenic artery in one case. Percutanous transluminal coronary angioplasty was followed by gastrectomy in one case. Ten patients had early and 16 had advanced gastric cancers. Among the 10 early gastric cancers, 6 were found within 1.5 years after CABG.

## Discussion

Successful gastrectomy after CABG using a RGEA depends on a good balance between safety and curability. Critical complications caused by handling of the RGEA while dissecting the lymph nodes should be avoided without compromising curative lymph node dissection. To avoid the potentially fatal coronary events, we performed safe handling, including preparations for inadvertent injuries and prevention of vessel spasm was performed, together with adequate monitoring of systemic circulation.

To avoid graft injuries, it is important to clearly understand how to harvest and where to place the graft to the first CABG. Two major ways of harvesting have been employed, such as a pedicled graft that is harvested with surrounding soft tissues and a skeletonized graft that is harvested without them. A pedicled graft seemed to be popular, but recently a skeletonized graft has been employed. A pedicled graft is unlikely to be distinguished from surrounding tissues. On the other hand, a skeletonized graft is easily recognized because of the absence of adhesions to the abdominal wall or other structures [10,11]. The RGEA in case 2 was difficult to recognize because of severe adhesion to the abdominal wall and was identified by recognition of arterial pulsation around the duodenum, even though a skeletonized graft was employed. However, a skeletonized graft is recommended because it is easy to get an adequate length of the graft as well as to observe the graft directly [12]. For the RGEA graft, two routes have been generally used, such as an ante-gastric and a retro-gastric route [13,14]. Most cardiac surgeons prefer the ante-gastric route because it allows them to identify bleeding sites of the graft easily [15]. On the other hand, it causes adhesion to the anterior abdominal wall or the greater omentum; a complication that is not found with the retro-gastric route [14]. In any case, a careful abdominal incision below the umbilicus is recommended in either case if adhesion of the RGEA to the surrounding tissues is anticipated.

Graft injuries may suddenly cause coronary failure and fatal arrhythmia [3-8]. Adequate intraoperative preparation for such emergencies is, therefore, required. Before laparotomy, an arterial catheter was inserted in the right femoral artery for an artificial circulation in both of our cases, while the hepatic and splenic arteries were prepared during the operation for a possible re-anastomosis with the RGEA. Stretching of the RGEA may also cause similar complications. Indeed, it has been reported that stretching of the abdominal wall depressed the ST segment of ECG [6]. It is therefore, important to keep these unexpected complications in mind during the operation.

It has been reported that mechanical stimulations can easily induce spasm in arterial grafts [6,8]. Therefore, prevention of spasm in the RGEA graft during the operation is important [6,16]. We sprinkled papaverine hydrochloride around the RGEA in both cases and could carry out the operation safely without the RGEA spasm.

Early detections and adequate treatments for injuries are important to avoid critical complications [6,16]. Careful intraoperative monitoring using ECG, transesophageal echocardiography, Swan-Gantz catheter etc. are, therefore, necessary. As the extent of coronary failure depends on how much blood a graft vessel supplies to a coronary artery when complications occur [17], patency of a graft should be evaluated preoperatively by angiography, which will also show the location of the graft.

**Figure 5:**
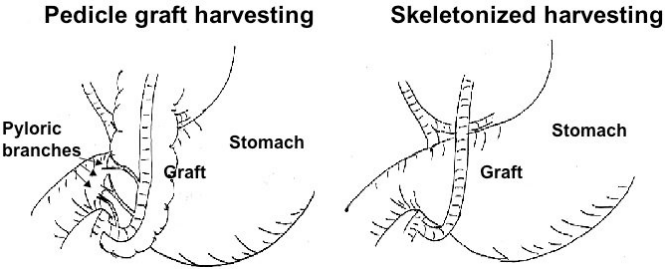
A schema of two harvesting methods. Pyloric branches of the RGEA are preserved in a pedicled graft harvesting (left), while they can be interrupted by a skeletonized graft harvesting (right).

Curative potential of this operation depends on the completeness of lymph node dissection around the RGEA graft, which are categorized as No.6 lymph node by the Japanese classification of gastric carcinoma [9]. Complete dissection by resecting the RGEA at its base requires an additional alternative graft, which makes the operation more complicated and results in prolongation of the procedure. Moreover, an alternative graft may itself cause lethal complications. Therefore, suitable patients requiring complete dissection because of high probability of the No.6 lymph node metastasis should be selected based on the location, depth of tumor invasion, pathological type etc [9]. Post-operative interval from CABG could also help in determining the strategy regarding the No.6 lymph node. Gastric cancers, occurring late after CABG, might have altered lymphatic flow. If the RGEA graft was harvested by the skeletonization method and freed up to its base with resections of pyloric branches during the first CABG operation (Fig 5), the No.6 lymph node metastasis would be very rare. Consequently, dissection of the No.6 lymph node would be unnecessary [3,18,19]. Among the 26 reviewed cases, No.6 lymph node dissection was performed in 16. Two out of 4 patients (50%) had metastasis to this lymph node within 12 months after CABG. Four out of 12 cases (33%) had this metastasis more than 12 months after CABG and 1 out of 5 cases (20%) had it more than 36 months after CABG. One case with cancer invasion of the submucosal region, had metastasis to the No.6 lymph node within 12 months after CABG. On the other hand, no metastasis to this node was seen more than 12 months after CABG in 6 cases where the cancer invaded the proper muscle region. Considering the pathological types of the cancer would also be important because all out of 6 cases with No.6 lymph node metastasis was poorly differentiated carcinomas. Therefore, No.6 lymph node dissection could be done away with for differentiated adenocarcinomas invading the submucosal or proper muscle region of the stomach and occurring more than 12 months after CABG with the skeletonization method. The cases having a low probability of the No.6 lymph node metastasis with or without the skeletonization method could also be spared so as to retain a good balance between safety and curability if intra-operative findings suggest no lymph node metastasis.

According to the database of the Cancer Institute Hospital (1946–2004) [20], undifferentiated gastric cancers, which occur in L or LD area and invade the submucosal region, metastasize to No. 6 lymph node at a very high rate of 23.6%. As the cancer in case 1 occurred 65 months after CABG, lymphatic flow was considered to be altered or interrupted. Thus, lymph node dissection was thought unnecessary. However, we were not sure as to whether pyloric branches of gastroepiploic artery still persisted. So we dissected around the No.6 lymph node so far as we could do safely and easily for intra-operative frozen section examination. However, it is unclear whether the manipulation for sampling of the nodes is safe and whether frozen section examination is enough for the negative diagnosis of lymph node metastasis. From that point of view, sampling of the nodes should be restricted to cases that are strongly suspected to have the No.6 lymph node metastasis such as the cases with swollen lymph nodes around the base of the RGEA or multiple metastasis of other lymph nodes. According to the database of the Cancer Institute Hospital (1946–2004) [20], gastric cancers having selective metastasis to the No.6 lymph node are seen in 1.6% of 12,000 gastric cancer cases. In case 1, the operation should have been completed by isolation of the graft without sampling of the nodes. As the cancer in case 2 occurred 3 months after the CABG, original lymph flow was considered to have persisted. Therefore, the No.6 lymph node dissection was thought necessary. However, undifferentiated gastric cancers, which occur in the LM/M/ML area and invade the mucosa, metastasize to No.6 node at a low rate of 0.6% according to the database of the Cancer Institute Hospital (1946–2004) [20]. Thus, the operation in case 2 should also have been completed by isolation of the graft without sampling of the nodes like that in case 1.

Among the 10 cases having early gastric cancers in reviewed 26 cases, 6 were found within 1.5 years after CABG. These cases could have been diagnosed at an even earlier stage by using a gastric fiberscopic examination before and after CABG, which could give them opportunities to receive a less invasive operation such as endoscopic mucosal resection. Three advanced cases detected within 1.5 years after CABG should have been found during the first CABG.

## Conclusion

Adequate intraoperative care as well as an optimal lymph node dissection considering a graft harvesting method at the first CABG may lead to successful gastrectomy after CABG using a RGEA. Therefore, this operation should be carried out with careful management by both gastrointestinal and cardiovascular surgeons.

## Abbreviations

RGEA: right gastroepiploic artery,

CABG: coronary artery bypass grafting,

pN: pathological lymph node metastasis,

pT: pathological tumor invasion,

## Competing interests

The author(s) declare that they have no competing interests.

## Authors' contributions

**YK **designed the study and participated in the writing process. **KS **designed the study, carried out the data and picture acquisition as well as bibliographic research, drafted and revised the manuscript. **HW, HAB, TK and KY **participated in manuscript revision process. **HW, HO, YS and TK **they participated in the editing process. All authors read and approved the final manuscript.

## References

[B1] Suma H, Wanibuchi Y, Terada Y, Fukuda S, Takayama T, Furuta S (1993). The right gastroepiploic artery graft. Clinical and angiographic midterm results in 200 patients. J Thorac Cardiovasc Surg.

[B2] Lytle BW, Cosgrove DM, Ratliff NB, Loop FD (1989). Coronary artery bypass grafting with the right gastroepiploic artery. J Thorac Cardiovasc Surg.

[B3] Takahashi T, Sawai S, Ogihara A, Yamaguchi T, Nakagawa N, Okano S, Takahashi S (1993). An adequate Surgery for gastric cancer in view of lymph flows. Shoukaki Geka.

[B4] Uchida N, Kawakami K, Kaku S, Kawaguchi M, Nakamitsu A, Fujii T (1996). Five cases of gastric cancer occurred after coronary artery bypass grafting using the right gastroepiploic artery. Shoukaki Geka.

[B5] Uchida N, Kawaue Y (1995). Upper abdominal complications after coronary artery bypass operations using right gastroepiploic artery. J Jpn Assn Thorac Surg.

[B6] Tanaka M, Terada Y, Sakamoto M (1994). Problems of cholecystectomy for acute cholecystitis after coronary artery bypass grafting using the right gastroepiploic artery. Geka.

[B7] Sugimoto M, Hasagawa H, Ogiso S, Sakamoto E, Igami T, Mori T (2002). Distal gastrectomy in a patient with an in situ right gastroepiploic artery graft. Rinshou Geka.

[B8] Yamabuki K (1997). Thickness of the muscle layer of the gastroepiploic artery and the internal mammary artery-a presumable factor of flow instability in GEA during the perioperative period. J Jpn Assn Thorac Surg.

[B9] Japanese, Gastric, Cancer, Association (1998). Japanese classification of gastric carcinoma – 2nd English edition.

[B10] Koto Kea (2003). The method of skeletonized right gastroepiploic artery is useful for following upper abdominal cancer operation. Kyobu Geka.

[B11] Yunoki J (2004). Skeletonized right gastroepiploic artery for coronary artery bypass grafting; Evaluation of intraoperative graft flow and postoperative angiographic result. Kyoubu Geka.

[B12] Gagliardotto P, Coste P, Lazreg M, Dor V (1998). Skeletonized right gastroepiploic artery used for coronary artery bypass grafting. Ann Thorac Surg.

[B13] Pasic M, Carrel T, Segesser LV, Turina M (1994). Postoperative diaphragmatic hernia after use of the right gastroepiploic artery bypass grafting. J Thorac Cardiovasc Surg.

[B14] Charles A, John E, John C (1995). Laparotomy after using the gastroepiploic artery graft: Retrogastric versus antegastric route. Ann Thorac Surg.

[B15] Shimizu J, Hirano Y, Kinoshita S, Tatsuzawa Y, Kawaura Y, Takahashi S (2004). Gastric cancer occurred after coronary artery bypass grafting using the right gastroepiploic artery. Ann Thorac Cardiovasc Surg.

[B16] Shirota S, Kajiyama Y, Iwanuma Y, Tomita N, Amano T, Isayama F, Tsurumaru M (2006). Three cases of advanced gastric cancer after coronary artery bypass graft using the right gastroepiploic artery. Shujutu.

[B17] Shikano T, Koshikawa K, Sawazaki M, Kiriyama K, Wada M, Taniguchi K, Suenaga H (2006). Three cases of gastrectomy for gastric cancer after coronary artery bypass grafting using the right gastroepiploic artery. Jpn J Gastroenterol Surg.

[B18] Tanaka K, Miyairi T, Matsumoto J, Murakawa T, Mizuno A, Saitoh H (1995). A case of subtotal gastrectomy for gastric cancer and cholecystectomy with preservation of the right gastroepiploic artery graft used for coronary artery bypass grafting. Jpn J CardiovascSurg.

[B19] Hayashi S, Kawaue Y, Sueshiro M, Kado S, Ono Y (1994). A case of gastric cancer occurred after coronary artery bypass grafting using the right gastroepiploic artery. J Jpn Assn Thorac Surg.

[B20] Nakajima T, Yamaguchi T (2006). Ganken igan database 1946–2004 (Cancer Institute Hospital gastric cancer database 1946–2004).

[B21] Hashiguchi N, Kubota T, Otani Y, Yoshida M, Maeda S, Tokuyama J, Wada N, Suganuma K, Kuwano Y, Kumai K (2004). Surgery for advanced gastric cancer after coronary artery bypass grafting using the right gastroepiploic artery: report of a case. Surg Today.

[B22] Shinhara R, Yamane S, Matsumoto T, Hukuma T, Sanada O (2000). Two cases of gastrectomy after coronary artery bypass grafting using the right gastroepiploic artery. Shujutsu.

[B23] Perrault LP, Rheault MJ, Carrier M (1994). Total gastrectomy in a patient with an in situ right gastroepiploic artery graft. Ann Thorac Surg.

[B24] Yamada T, Kitagawa S, Nakagawa M, Tsubota M, Seki M (1999). A case of gastric cancer after coronary artery bypass grafting with the gastroepiploic artery. Rinsho Geka.

[B25] Tomimoto S, Hashidume T, Hatano T, Nishio K (1997). A case of total gastrectomy for gastric cancer after coronary artery bypass grafting using the gastroepiploic artery. Jap Cir J.

[B26] Suzuki K, Kobayashi Y, Miyata K, Yoneyama F (2004). A case of distal gastrectomy for advanced gastric cancer preserving the right gastroepiploic artery graft for coronary bypass. J Jpn Surg Assoc.

